# Response to Prone Position in COVID-19 and Non-COVID-19 Patients with Severe ARDS Supported by vvECMO

**DOI:** 10.3390/jcm12123918

**Published:** 2023-06-08

**Authors:** Laura Textoris, Ines Gragueb-Chatti, Florence Daviet, Sabine Valera, Céline Sanz, Laurent Papazian, Jean-Marie Forel, Sami Hraiech, Antoine Roch, Christophe Guervilly

**Affiliations:** 1 Service de Médecine Intensive Réanimation, Hôpital Nord, Assistance Publique-Hôpitaux de Marseille, 13015 Marseille, France; laura.textoris@ap-hm.fr (L.T.); ines.gragueb-chatti@ap-hm.fr (I.G.-C.); florence.daviet@ap-hm.fr (F.D.); sabine.valera@ap-hm.fr (S.V.); celine.sanz@ap-hm.fr (C.S.); laurent.papazian.pro@gmail.com (L.P.); jean-marie.forel@ap-hm.fr (J.-M.F.); sami.hraiech@ap-hm.fr (S.H.); antoine.roch@ap-hm.fr (A.R.); 2 Centre Hospitalier de Bastia, Service de Réanimation, 604 Chemin de Falconaja, 20600 Bastia, France; 3 Centre d’Études et de Recherches sur les Services de Santé et Qualité de vie EA 3279, Aix-Marseille Université, 13005 Marseille, France

**Keywords:** COVID-19, severe ARDS, venovenous ECMO, prone position, respiratory system compliance

## Abstract

Background: For moderate to severe acute respiratory distress syndrome (ARDS), lung-protective ventilation combined with prolonged and repeated prone position (PP) is recommended. For the most severe patients for whom this strategy failed, venovenous extracorporeal membrane oxygenation (vv-ECMO) allows a reduction in ventilation-induced lung injury and improves survival. Some aggregated data have suggested a benefit regarding survival in pursuing PP during vv-ECMO. The combination of PP and vv-ECMO has been also documented in COVID-19 studies, although there is scarce evidence concerning respiratory mechanics and gas exchange response. The main objective was to compare the physiological response of the first PP during vv-ECMO in two cohorts of patients (COVID-19-related ARDS and non-COVID-19 ARDS) regarding respiratory system compliance (C_RS_) and oxygenation changes. Methods: This was a single-center, retrospective, and ambispective cohort study in the ECMO center of Marseille, France. ECMO was indicated according to the EOLIA trial criteria. Results: A total of 85 patients were included, 60 in the non-COVID-19 ARDS group and 25 in the COVID-19-related ARDS group. Lung injuries of the COVID-19 cohort exhibited significantly higher severity with a lower C_RS_ at baseline. Concerning the main objective, the first PP during vv-ECMO was not associated with a change in C_RS_ or other variation in respiratory mechanic variables in both cohorts. By contrast, oxygenation was improved only in the non-COVID-19 ARDS group after a return to the supine position. Mean arterial pressure was higher during PP as compared with a return to the supine position in the COVID-19 group. Conclusion: We found distinct physiological responses to the first PP in vv-ECMO-supported ARDS patients according to the COVID-19 etiology. This could be due to higher severity at baseline or specificity of the disease. Further investigations are warranted.

## 1. Introduction

Acute respiratory distress syndrome (ARDS) is an acute respiratory failure that is classified into three stages of severity according to the Berlin definition [1]. For moderate-to-severe ARDS, lung-protective ventilation which includes a low tidal volume (V_t_)–low plateau pressure (P_plat_) ventilation strategy combined with prolonged and repeated prone position (PP) is recommended [2]. 

Venovenous extracorporeal membrane oxygenation (vv-ECMO) allows decreasing V_t_, airway inspiratory pressures, and the respiratory rate (RR), which all individually can induce or worsen ventilator-induced lung injuries (VILIs) [3,4]. For the most severe ARDS patients for whom the combination of lung-protective ventilation combined with PP failed, the early initiation of vv-ECMO increased survival [5]. 

In addition, retrospective aggregated data suggest a potential benefit of continuation or initiation of PP in vv-ECMO patients.

In December 2019, a new virus emerged in the region of Wuhan in China, the severe acute respiratory syndrome coronavirus 2 (SARS-CoV-2) which was responsible for the global pandemic of coronavirus disease 2019 (COVID-19) [6]. Although most patients infected by COVID-19 present mild or moderate symptoms, about 10% will need hospitalization and 1.5% will require intensive care unit (ICU) hospitalization. Among them, around 70% need respiratory support for acute respiratory failure. vv-ECMO has been increasingly used during the first wave of the pandemic and thereafter [7]. 

Interestingly, some observational cohorts report a very high rate of PP use (up to 70–90%) during vv-ECMO [8,9,10]. 

Therefore, the aim of the study was to compare the physiological response of PP between two cohorts of severe ARDS patients (COVID-19-related ARDS and non-COVID-19 ARDS) supported by vv-ECMO.

## 2. Materials and Methods 

### 2.1. Study Design and Ethics Approval

We performed a single-center, retrospective, and ambispective cohort study. The study protocol was reviewed and approved by the Marseille Teaching Hospital Institutional Review Board (PADS21-89) and by the ethics committee of the French intensive care society (CE SRLF 21-47). According to French law, informed consent was not required due to the design of the study, and we only collected the non-opposition form from the patient or their surrogate.

### 2.2. Study Settings 

All patients included were in a tertiary university hospital in Marseille, France. Patients were cannulated either directly in the department or in another ICU in the Provence-Alpes-Côtes-d’Azur region and immediately transferred by the vv-ECMO mobile retrieval team [11]. 

### 2.3. Population

The non-COVID-19 cohort was built from a previous study [12]. Only patients with available physiological data were included in the cohort. The ambispective cohort included consecutive COVID-19 patients hospitalized between 1 January 2021 and 31 December 2021 and supported by vv-ECMO. The first patient was included on 2 February 2021, and the last patient was included on 11 November 2021. 

vv-ECMO was indicated according to the EOLIA trial criteria, either refractory hypoxemia defined by a ratio of partial pressure of arterial oxygen to fraction of inspired oxygen (PaO_2_:FiO_2_ ratio) < 50 mmHg for at least 3 h or a PaO_2_:FiO_2_ ratio < 80 mmHg for at least 6h despite a FiO_2_ ≥ 80% and a positive end-expiratory pressure (PEEP) ≥ 10 cm H_2_O, or respiratory acidosis with arterial blood pH < 7.25 with a partial pressure of arterial carbon dioxide (PaCO2) > 60 mmHg for > 6 h (with RR increased to 35 cycles/minute) resulting from mechanical ventilation settings adjusted to keep P_plat_ ≤ 32 cm H_2_O (first, V_t_ reduction by 1 mL/kg decrements to 4 mL/kg; then, PEEP reduction to a minimum of 8 cm H_2_O) [5]. 

### 2.4. Primary and Secondary Endpoints

The primary endpoint was the change in the respiratory system compliance and oxygenation between the start and the end of the first PP session during vv-ECMO in the two cohorts (COVID-19 and non-COVID-19 patients).

Secondary endpoints were the changes in other respiratory mechanics variables, arterial blood gas and ECMO settings during the same time frame, safety assessment of the first PP, and clinical outcomes in the two cohorts.

### 2.5. vv-ECMO Management 

All the patients were cannulated using a percutaneous approach. The oxygen fraction delivered by the membrane oxygenator (FmO_2_, %) was set at 100. Then, the sweep gas flow was progressively increased to reach an arterial pH value above 7.30. The vv-ECMO blood flow was progressively increased to obtain a pulsed oxygen saturation (SpO_2_) > 90% (or PaO_2_ > 60 mmHg) and to reach at least 60 % of the actual cardiac output. Anticoagulation with intravenous unfractionated heparin was used to target an anti-Xa activity between 0.3 and 0.6 IU/mL. The triggering limit for transfusion was 8 g/dL for hemoglobin, 50 Giga/L for platelet, and 1.5 g/L for fibrinogen. Hemolysis was also investigated daily during the vv-ECMO run.

### 2.6. Mechanical Ventilation Protocol during vv-ECMO

Volume-controlled with constant flow mode was first used. V_t_ was set to obtain a maximum P_plat_ of 25 cm H_2_O while PEEP was kept above 10 cm H_2_O. RR was decreased between 10 and 15 cycles/min. Continuous perfusion of neuromuscular blockers was pursued for 48 h after cannulation. 

In case of the early improvement of respiratory function or after 48 h, a switch to partial assisted pressure-controlled mode as airway pressure release ventilation (APRV) or bi-level positive airway pressure (Bi-PAP) was encouraged after interruption of neuromuscular blockers. 

### 2.7. Prone Position Procedure 

All included patients received at least one 16 h session of PP during the vv-ECMO run. The ICU team followed a written protocol for each maneuver including eye occlusion protection and protection of skin from all catheters and invasive devices (vv-ECMO cannulas, tubing, thoracic drain, and bladder probe). The intensivists in charge of the patient stood at the head to hold the intubation tube and jugular cannula in place. Two people stood on either side of the patient. A fifth person secured vv-ECMO tubing and prevented any dislodgment of vv-ECMO cannulas. Two specific air mattresses were then placed on the patient’s head, thorax, and hips to prevent pressure sores.

### 2.8. Data Collection 

Demographics (gender, age, weight, height, BMI, comorbidities) and severity scores were recorded at the inclusion. 

Before vv-ECMO, data on duration of mechanical ventilation, worse PaO_2_:FiO_2_ ratio, use and number of PP sessions, administration of inhaled nitric oxide (iNO), and eventual renal replacement therapy were collected.

The date of cannulation, vv-ECMO configuration, and number of PP sessions on vv-ECMO were also recorded. We computed the duration of vv-ECMO, vv-ECMO weaning rate, and ICU and hospital mortality rates as outcomes.

Concerning respiratory mechanics variables, we recorded V_t_ (mL), RR (cycles/min), minute ventilation (V_M_, L/min), PEEP (cm H_2_O), peak inspiratory pressure (P_peak_, cm H_2_O), and FiO_2_ (%) for each patient. At the same time, we measured P_plat_ (cm H_2_O) by using an inspiratory pause (1 s) and calculated the compliance of the respiratory system (C_RS_, mL/cm H_2_O) by dividing V_t_ by the difference between P_plat_ and total PEEP, measured by using an expiratory pause (5 s), also called driving pressure (ΔP = P_plat_ − PEEP_total_, cm H_2_O). Mechanical power (MP, J/min) was only available in COVID-19 patients and was calculated as follows:

$$MP=0.098\times Vt\left(L\right)\times RR(c/min)\times \left(Ppeak-\frac{\Delta P}{2}\right)(cm\;H_{2}O)$$

with 0.098 the conversion factor from L/cm H_2_O to joules [13].

For the COVID-19 ambispective cohort, we collected additional data. One hour before (H-1 PP) and one hour after the PP (H+1 PP), and one hour before the supine position (H-1 SP) and one hour after (H+1 SP), we recorded hemodynamic parameters (heart rate and mean arterial pressure), arterial blood gas (pH, PaO_2_, PaCO_2_, saturation of arterial oxygen (SaO_2_), PaO_2_:FiO_2_ ratio), ventilator parameters (V_t_, RR, PEEP, P_peak_, P_plat_, V_M_, FiO_2_, and C_RS_), and vv-ECMO parameters (vv-ECMO blood flow, sweep gas, and FmO_2_). 

### 2.9. Assessment of Safety of Prone Position 

In the COVID-19 cohort, we recorded and compared pre-specified adverse events potentially associated with PP maneuvers, including severe hypoxemia (SpO_2_ < 80% for at least 5 min), decrease in vv-ECMO blood flow > 20% of baseline; mean arterial pressure < 55 mmHg for at least 5 min; pneumothorax; tracheal tube obstruction; and vv-ECMO cannula, intravenous catheter, or endotracheal tube dislodgment. 

### 2.10. Statistical Analysis

No sample size was calculated. However, we planned to include 25 patients in the COVID-19 ambispective cohort. For the non-COVID-19 retrospective cohort, we extracted available data of interest from a previous study [12]. 

Qualitative variables were expressed as numbers and percentages. Comparisons between groups were performed with the chi^2^ test or Fisher test as appropriate.

Quantitative variables were expressed as median (interquartile range) or mean ± standard deviation. Comparisons between groups were performed with the U Mann–Whitney test or the Student t test as appropriate.

Comparisons between times were performed with the Kruskal–Wallis test or with ANOVA as appropriate. Post hoc tests were performed with the Tukey and Bonferroni tests.

A *p* value < 0.05 was considered as significant.

All statistics were calculated and figures were created with SPSS 20.0 (IBM, Armonk, NY, USA).

## 3. Results

Eighty-five patients were included, 60 in the non-COVID-19 ARDS cohort and 25 in the COVID-19 ARDS cohort.

### 3.1. Baseline Characteristics and Outcomes 

The baseline characteristics of the two cohorts are displayed in Table 1. 

Besides obvious differences in ARDS etiology, the non-COVID-19 ARDS cohort had higher severity scores and less frequently received adjunctive therapy (PP or iNO) before vv-ECMO implantation as compared with the COVID-19 ARDS group. 

In the COVID-19 ARDS cohort, 12 patients (48%) had thoracic CT scans realized at ECMO initiation. The percentage of lung consolidation was 75 (55–90)%.

Concerning the pre-specified outcomes, there was no difference in the vv-ECMO duration, vv-ECMO weaning rate, ICU mortality, and hospital mortality between the two groups.

First PP was considered after a median of 4 days of vv-ECMO. This delay was shorter in the COVID-19 ARDS cohort as compared with the non-COVID-19 ARDS cohort. 

### 3.2. Effects of the First PP under vv-ECMO in the COVID-19 ARDS Group

**Figure 1:**
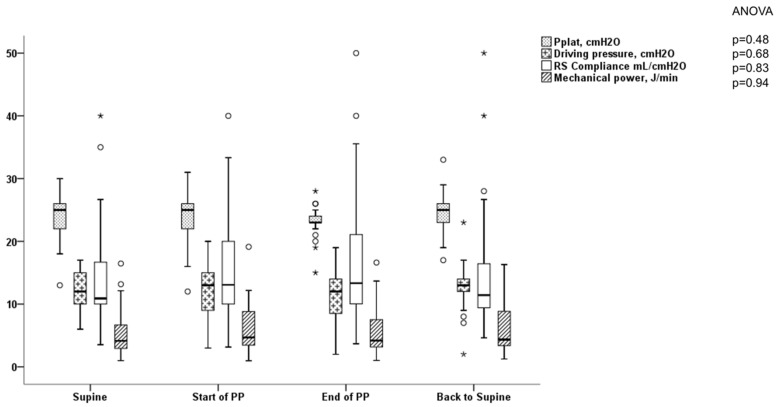
Variation in respiratory mechanics parameters during the first PP under vv-ECMO in patients with COVID-19 ARDS. The empty circles represent the outliers and the black stars represent the extreme values.

**Figure 2:**
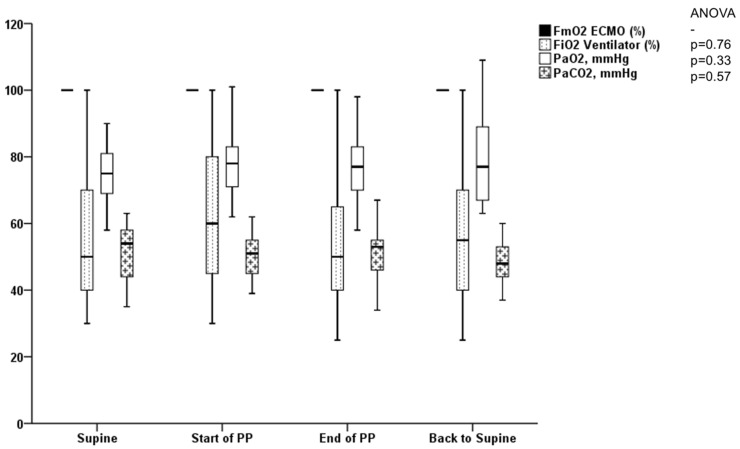
Variation in gas exchange during the first prone positioning under vv-ECMO for COVID-19 ARDS.

No significant effect was observed among the respiratory mechanics variables, the vv-ECMO settings, and gas exchanges during the first PP under vv-ECMO (Table 2, Figure 1 and Figure 2). Concerning hemodynamics, we found a significant variation in mean arterial pressure with an increase during PP.

Definition of abbreviations and formula: Pplat = plateau airway pressure; RS compliance = respiratory system compliance calculated by tidal volume divided by driving pressure; mechanical power calculated by the simplified equation of Gattinoni (0.098 × tidal volume (L) × respiratory rate (cycles/min) × peak inspiratory pressure less driving pressure divided by 2); PP = prone position.

Definition of abbreviations: FmO_2_ = oxygen fraction delivered by the membrane oxygenator of the vv-ECMO; FiO_2_: oxygen fraction inspired delivered by the ventilator; PaO_2_ = partial pressure of arterial oxygen; PaCO_2_ = partial pressure of arterial carbon dioxide; PP = prone position.

### 3.3. Effects of the First PP under vv-ECMO in the Non-COVID-19 ARDS Group

Respiratory mechanics, vv-ECMO settings, and arterial blood gas before and after the first PP under vv-ECMO in the non-COVID-19 ARDS cohort are displayed in Table 3.

No significant change in respiratory mechanics was observed, whereas PaO_2_ and the PaO_2_:FiO_2_ ratio increased significantly from 75 ± 14 mmHg to 84 ± 22 mmHg (*p* = 0.02) and from 135 ± 57 mmHg to 176 ± 72 mmHg (*p* = 0.001), respectively. We performed a sensitivity analysis restricted to the non-COVID-19 cohort who received PP before ECMO (N = 44) and found no difference except for a slight decrease in ECMO blood flow after the first PP (4 ± 0.9 L/min and 3.7 ± 1 L/min, *p* = 0.03). 

### 3.4. Comparison between COVID-19 ARDS Group and Non-COVID-19 ARDS Group before and after the First PP under vv-ECMO

Comparisons of respiratory mechanics, vv-ECMO settings, and arterial blood gas before and after the first PP under vv-ECMO between COVID-19 ARDS and non-COVID-19 ARDS are displayed in Table 4.

Before the first PP under vv-ECMO, PEEP and C_RS_ were higher in patients with non-COVID-19 ARDS as compared with patients with COVID-19 ARDS. Conversely, ∆P was lower in the non-COVID-19 ARDS cohort. These differences were consistent after the first PP.

In addition, no difference was observed for P_plat_ and Vt. A slightly higher respiratory rate was used in the COVID-19 ARDS cohort with no difference in minute ventilation. Before the first PP, higher sweep gas flow and RR resulting in lower PaCO_2_ were found in the non-COVID-19 ARDS cohort. These differences were also consistent after the first PP. A limited increase in C_RS_ in the non-COVID-19 ARDS group and a limited decrease in C_RS_ resulted in a significant difference in ΔP between groups after the first PP.

### 3.5. Assessment of Safety in the COVID-19 Cohort

Among pre-specified safety concerns, no patient presented a serious adverse event during the first PP under vv-ECMO.

## 4. Discussion 

In our retrospective and ambispective single-center cohort study, we observed distinct responses to the first PP in severe ARDS supported by vv-ECMO depending on COVID-19 etiology.

Whereas no significant difference among C_RS_ and other respiratory mechanics variables was observed, a significant increase in oxygenation parameters was ensured by PP only in the non-COVID-19 ARDS cohort.

vv-ECMO is a valuable therapeutic option for patients with very severe ARDS and refractory hypoxemia when a strategy associating lung-protective ventilation with low tidal volume and low plateau pressure associated with prolonged and repeated prone position fails [5]. 

While PP and vv-ECMO have been proven to individually decrease mortality, the combination of both has not been investigated in a randomized clinical study.

In our cohort of patients with non-COVID-19 ARDS, we found an improvement in oxygenation-related parameters after the first PP under vv-ECMO. The increase in PaO_2_ and PaO_2_:FiO_2_ ratio may be the result of an improvement of the ventilation/perfusion ratio by homogenization of transpulmonary pressures and decreasing lung strain rather than an increase in alveolar recruitment since we did not observe an increase in C_RS_. 

An increase in oxygenation during PP during vv-ECMO has been reported in a previous meta-analysis both in COVID and non-COVID-19 patients and seems consistent [14]. Despite a significant decrease in driving pressure, the global effect on C_RS_ was not significant.

This could be due to the delay in proning the patient during ECMO. Indeed, Giani et al. found that non-COVID-19 ARDS patients who were proned after 5 days of vv-ECMO start did not improve in C_RS_ despite improvement in oxygenation [15].

Despite a shorter delay in proning the patients in the COVID-19 cohort, it was not associated with improvement in oxygenation or C_RS_. Our COVID-19 ARDS cohort had notably a lower C_RS_ but similar oxygenation severity compared to the non-COVID-19 ARDS cohort. We cannot exclude that those patients had a higher degree of secondary lung fibrosis limiting the beneficial effects of the prone position [16].

In addition, the assessment of the first PP under vv-ECMO may be insufficient to demonstrate an effect on oxygenation and/or on C_RS_.

A positive effect of PP has been demonstrated after the repetition of sessions regardless of the effect on oxygenation [17]. Therefore, we can hypothesize the potential protective effects of PP on ventilator-induced lung injuries at a non-clinically measurable level.

Contrary to the hypothesis raised at the beginning of the pandemic, large studies and a systematic review have demonstrated that C_RS_ measured close to the time of the initiation of invasive mechanical ventilation was normally distributed [18,19] and was comparable to that in non-COVID-19 ARDS patients [20]. This does not support the concept of distinct phenotypes in COVID-19-related ARDS. Finally, in the late stage of the disease (from the third week), the likelihood of oxygenation improving with prone positioning becomes extremely low [20,21,22]. 

No major complication related to PP during vv-ECMO was reported in our study. In the cohort of COVID-19 patients, a significant increase in mean arterial pressure in the PP position was observed. This effect may be related to an increase in venous return and mean systemic pressure [23].

One hundred percent of the COVID-19 cohort but only 74% of the non-COVID-19 cohort had a first PP attempt before ECMO implementation. This could be also taken into account regarding the lack of response for the COVID-19 cohort. 

Several limitations in our study should be noted. First, due to the design of the study, a significant proportion (36%) of the non-COVID-19 cohort with missing respiratory mechanics variables or gas exchange data was not included. In addition, we included a relatively small sample size in the COVID-19 cohort to minimize the missing data. Therefore, the risk of type II error should be mentioned. Second, the decision to perform or not perform PP was at the discretion of the medical team in charge. No threshold for the PaO_2_:FiO_2_ ratio (which is difficult to interpret during vv-ECMO) was determined in the design of the protocol. It cannot be ruled out that a number of PP sessions were performed as a rescue therapy and not routinely when the PaO_2_:FiO_2_ ratio was below 150 mmHg, which may, at least partly, explain the non-significance of the study. Third, the COVID-19 variants during successive surges may have played a role in response to PP. Finally, the possible beneficial effect of pursuing PP during vv-ECMO on vv-ECMO duration or mortality reported in a very recently terminated randomized clinical trial [24] needs urgent confirmation.

## 5. Conclusions

We did not observe changes in C_RS_ during the first PP performed in two distinct cohorts of ARDS patients supported by vv-ECMO. In non-COVID-19 patients, PP was associated with improvement in oxygenation. We cannot exclude beneficial effects at a non-clinical level (e.g., on biotrauma), and these effects need further investigation.

## Figures and Tables

**Table 1:** Baseline characteristics of the two cohorts.

	COVID-19 ARDS	Non-COVID-19 ARDS	*p* Value
	N = 25	N = 60	
Age, median (IQR)	55 (45–61)	51 (38–64)	0.79
Male sex, n (%)	18 (72)	44 (74)	0.80
Body mass index (kg/m^2^), median (IQR)	30 (27.6–35.2)	28.7 (25.5–35.4)	0.38
SAPS 2 at admission, median (IQR)	41 (31–49)	47 (42–55)	0.006
SOFA score at inclusion, median (IQR)	7 (4–9)	10 (8–12)	0.001
Cause of ARDS			
COVID-19 Viral non-COVID-19 Bacterial Aspiration Pulmonary—others Extrapulmonary sepsis	25 (100) 0 (0) 0 (0) 0 (0) 0 (0) 0 (0)	0 (0) 13 (22) 35 (58) 2 (3.5) 8 (13) 2 (3.5)	<0.001
Comorbidity, n (%)			
Immunocompromised Hypertension Diabetes mellitus Chronic renal failure Chronic obstructive pulmonary disease	3 (12) 11 (44) 4 (16) 1 (4) 7(28)	0 (0) 14 (24) 8 (14) 2 (3.5) 11 (19)	0.07 0.06 0.77 0.89 0.34
Before vv-ECMO			
Duration of mechanical ventilation, median (IQR) Prone position, n (%) Inhaled nitric oxide, n (%) PaO_2_:FiO_2_ ratio, mmHg, median (IQR) Renal replacement therapy, n (%)	5 (1–7) 25 (100) 20 (80) 68 (50–74) 1 (4)	3 (1–7) 44 (74) 26 (44) 66 (50–81) 2 (3.5)	0.47 0.005 0.002 0.93 0.89
Referred from other ICUs, n (%) Retrieved by vv-ECMO mobile team, n (%)	24 (96) 21 (84)	56 (95) 48 (81)	0.83 0.77
vv-ECMO configuration, n (%)			
Femoro-jugular Femoro-femoral Jugulo-jugular	25 (100) 0 (0) 0 (0)	55 (92) 4 (7) 1 (1)	0.32
Outcomes			
ECMO days before PP, median (IQR) Number of PP sessions on vv-ECMO, median (IQR) vv-ECMO duration, days, median (IQR) vv-ECMO weaning rate, n (%) ICU mortality rate, n (%) Hospital mortality rate, n (%)	2 (1–3) 4 (3–6) 23 (15–34) 18 (72) 12 (48) 12 (48)	5 (3–7) 2 (1–4) 20 (13–36) 38 (64) 32 (54) 36 (61)	<0.001 <0.001 0.75 0.50 0.60 0.27

Definition of abbreviations: IQR = interquartile range; SAPS 2 = simplified acute physiology score; SOFA = sequential organ failure assessment score; ARDS = acute respiratory distress syndrome; COVID-19 = coronavirus disease 2019; vv-ECMO = venovenous extracorporeal membrane oxygenation; PP = prone position; PaO_2_:FiO_2_ ratio = ratio of the partial pressure of arterial oxygen to the fraction of inspired oxygen; ICU = intensive care unit.

**Table 2:** Evolution of respiratory mechanics, vv-ECMO settings, arterial blood gas, and hemodynamics during the first prone position in the COVID-19 ARDS cohort.

	Baseline Supine H-1 PP	Start of Prone H+1 PP	End of Prone H-1 SP	Return to Supine H+1 SP	*p* Value
Ventilatory parameters					
Tidal volume, mL, median (IQR)	150 (106–215)	145 (100–220)	150 (115–200)	160 (100–230)	0.97
Plateau airway pressure, cm H_2_O, median (IQR)	25 (21–26)	25 (22–26)	23 (23–24)	25 (22–26)	0.48
Peak inspiratory pressure, cm H_2_O, median (IQR)	27 (23–29)	29 (26–32)	26 (25–30)	29 (25–31)	0.36
PEEP, cm H_2_O, median (IQR)	12 (9–14)	12 (10–14)	12 (10–14)	12 (10–14)	0.93
Driving pressure, cm H_2_O, median (IQR)	12 (10–15)	13 (9–15)	12 (8–14)	13 (11–14)	0.68
Respiratory rate, cycles/min, median (IQR)	15 (13–17)	15 (13–16)	15 (12–16)	15 (13–19)	0.89
Minute ventilation, L/min, median (IQR)	2.2 (1.5–3.7)	2 (1.5–3.6)	2.1 (1.5–3.4)	2.4 (1.5–3.8)	0.94
Respiratory system compliance, mL/cm H_2_O, median (IQR)	11 (10–17)	13 (10–21)	13 (10–21)	11 (9–17)	0.83
Mechanical power, J/min, median (IQR)	4.1 (2.8–7.2)	4.7 (3.4–9)	4.2 (3.1–8.2)	4.3 (3.4–9)	0.94
Inspired fraction of oxygen, %, median (IQR)	50 (40–75)	60 (45–80)	50 (40–70)	55 (35–75)	0.76
vv-ECMO parameters					
vv-ECMO blood flow, L/min, median (IQR)	3.8 (3.3–4.7)	4 (3.3–4.4)	3.8 (3.2–4.8)	3.9 (3.2–4.6)	0.99
Sweep gas flow, L/min, median (IQR)	5 (3.5–6)	5 (4–6)	5 (3.5–7)	5 (3.7–6.5)	0.93
Membrane lung fraction of oxygen, %, median (IQR)	100 (100–100)	100 (100–100)	100 (100–100)	100 (100–100)	-
Arterial blood gas					
PaO_2_, mmHg, median (IQR)	75 (69–81)	78 (69–85)	77 (70–83)	77 (67–89)	0.33
PaCO_2_, mmHg, median (IQR)	54 (43–58)	51 (43–55)	53 (45–56)	48 (44–54)	0.57
PaO_2_:FiO_2_ ratio, mmHg, median (IQR)	140 (95–185)	142 (98–186)	144 (127–207)	147 (95–221)	0.74
pH, median (IQR)	7.40 (7.36–7.42)	7.42 (7.37–7.43)	7.40 (7.35–7.44)	7.42 (7.39–7.45)	0.11
Hemodynamic parameters					
Heart rate, bpm, median (IQR)	89 (71–116)	92 (73–111)	96 (76–105)	81 (70–105)	0.70
Mean arterial pressure, mmHg, median (IQR)	80 (73–90)	87 (80–100) *	87 (77–100) *	73 (67–86)	0.002

Definition of abbreviations and formula: IQR = interquartile range; PEEP = positive end-expiratory pressure; mechanical power calculated by the simplified equation of Gattinoni (0.098 × tidal volume (L) × respiratory rate (cycles/min) × peak inspiratory pressure less driving pressure divided by 2); vv-ECMO: venovenous extracorporeal membrane oxygenation; PaO_2_ = partial pressure of arterial oxygen; PaCO_2_ = partial pressure of arterial carbon dioxide; PaO_2_:FiO_2_ ratio = ratio of the partial pressure of arterial oxygen to the fraction of inspired oxygen; PP = prone position; SP = supine position. * *p* < 0.05 compared with return to supine with post hoc Tukey and Bonferroni tests.

**Table 3:** Evolution of respiratory mechanics, vv-ECMO settings, and arterial blood gas before and after the first prone position in the non-COVID-19 ARDS cohort (N = 60).

	Supine before Proning	Supine after Proning	*p* Value
Ventilatory parameters			
Tidal volume, mL, mean ± sd	206 ± 110	201 ± 99	0.79
Plateau airway pressure, cm H_2_O, mean ± sd	25 ± 4	25 ± 4	0.21
PEEP, cm H_2_O, mean ± sd	15 ± 3	15 ± 3	0.85
Driving pressure, cm H_2_O, mean ± sd	11 ± 4	10 ± 4	0.28
Respiratory rate, cycles/min, mean ± sd	14 ± 6	13 ± 5	0.79
Minute ventilation, L/min, mean ± sd	2.9 ± 2.1	2.8 ± 2.2	0.84
Respiratory system compliance, mL/cm H_2_O, mean ± sd	22.4 ± 12.3	22.5 ± 12.3	0.95
Inspired fraction of oxygen, %, mean ± sd	63 ± 22	54 ± 18	0.022
vv-ECMO parameters			
vv-ECMO blood flow, L/min, mean ± sd	4 ± 0.8	3.8 ± 0.8	0.35
Sweep gas flow, L/min, mean ± sd	6 ± 2	6 ± 2	0.90
Membrane lung fraction of oxygen, %, mean ± sd	100 ± 0	100 ± 0	-
Arterial blood gas			
PaO_2_, mmHg, mean ± sd	75 ± 14	84 ± 22	0.002
PaCO_2_, mmHg, mean ± sd	45 ± 10	43 ± 9	0.32
PaO_2_:FiO_2_ ratio, mmHg, mean ± sd	135 ± 57	176 ± 72	0.001

Definition of abbreviations: sd = standard deviation; PEEP = positive end-expiratory pressure; vv-ECMO: venovenous extracorporeal membrane oxygenation; PaO_2_ = partial pressure of arterial oxygen; PaCO_2_ = partial pressure of arterial carbon dioxide; PaO_2_:FiO_2_ ratio = ratio of the partial pressure of arterial oxygen to the fraction of inspired oxygen.

**Table 4:** Comparisons of respiratory mechanics, vv-ECMO settings, and arterial blood gas before and after the first prone position between COVID-19 ARDS and non-COVID-19 ARDS.

	Supine before Proning		*p* Value	Supine after Proning		*p* Value
	COVID-19 ARDS N = 25	Non-COVID-19 ARDS N = 60		COVID-19 ARDS N = 25	Non-COVID-19 ARDS N = 60	
Ventilatory parameters						
Tidal volume, mL, median (IQR)	150 (106–215)	170 (150–243)	0.08	160 (115–240)	170 (130–250)	0.31
Plateau airway pressure, cm H_2_O, median (IQR)	25 (21–26)	26 (23–28)	0.06	25 (22–26)	25 (22–26)	0.90
PEEP, cm H_2_O, median (IQR)	12 (9–14)	15 (12–18)	<0.001	12 (10–14)	15 (12–18)	0.002
Driving pressure, cm H_2_O, median (IQR)	12 (10–15)	10 (7–13)	0.06	13 (11–14)	9 (7–12)	0.001
Respiratory rate, cycles/min, median (IQR)	15 (13–17)	12 (10–15)	0.01	15 (13–19)	12 (10–15)	0.006
Minute ventilation, L/min, median (IQR)	2.2 (1.5–3.7)	2.1 (1.5–3.3)	0.84	2.4 (1.5–3.8)	2.0 (1.5–3.0)	0.44
Respiratory system compliance, mL cm H_2_O, median (IQR)	11 (10–17)	20 (12–31)	0.009	11 (9–17)	21 (13–30)	0.005
Inspired fraction of oxygen, %, median (IQR)	50 (40–75)	60 (40–80)	0.19	55 (35–75)	50 (40–60)	0.35
ECMO parameters						
vv-ECMO blood flow, L/min, median (IQR)	3.8 (3.3–4.7)	3.8 (3.2–4.6)	0.76	3.9 (3.2–4.5)	3.7 (3.2–4.5)	0.53
Sweep gas flow, L/min, median (IQR)	5 (3.5–6)	6 (5–7)	0.04	5 (4–6)	6 (5–7)	0.24
Membrane lung fraction of oxygen, %, median (IQR)	100 (100–100)	100 (100–100)	1	100 (100–100)	100 (100–100)	1
Arterial blood gas						
PaO_2_, mmHg, median (IQR)	75 (69–81)	71 (64–82)	0.42	77 (67–89)	77 (68–92)	0.66
PaCO_2_, mmHg, median (IQR)	54 (43–58)	43 (39–49)	0.006	48 (44–54)	42 (38–50)	0.008
PaO_2_:FiO_2_ ratio, mmHg, median (IQR)	140 (95–185)	127 (92–162)	0.27	147 (95–221)	160 (125–214)	0.31

Definition of abbreviations: IQR = interquartile range; PEEP = positive end-expiratory pressure; vv-ECMO: venovenous extracorporeal membrane oxygenation; PaO_2_ = partial pressure of arterial oxygen; PaCO_2_ = partial pressure of arterial carbon dioxide; PaO_2_:FiO_2_ ratio = ratio of the partial pressure of arterial oxygen to the fraction of inspired oxygen; ARDS = acute respiratory distress syndrome; COVID-19 = coronavirus disease 2019.

## Data Availability

The datasets used and/or analyzed during the current study are available from the corresponding author on reasonable request.

## Abbreviations

APRV	airway pressure release ventilation
ARDS	acute respiratory distress syndrome
BMI	body mass index
COVID-19	coronavirus disease 2019
C_RS_	compliance of respiratory system
ΔP	driving pressure
FiO_2_	fraction of inspired oxygen
FmO_2_	oxygen fraction delivered by the membrane oxygenator
ICU	intensive care unit
iNO	inhaled nitric oxide
MP	mechanical power
PaCO_2_	partial pressure of arterial carbon dioxide
PaO_2_	partial pressure of arterial oxygen
PaO_2_:FiO_2_ ratio	ratio of partial pressure of arterial oxygen to fraction of inspired oxygen
PEEP	positive end-expiratory pressure
PP	prone position
P_peak_	peak inspiratory pressure
P_plat_	plateau pressure
RR	respiratory rate
SARS-CoV-2	severe acute respiratory syndrome coronavirus 2
SP	supine position
SaO_2_	saturation of arterial oxygen
VILI	ventilator-induced lung injury
V_M_	minute ventilation
V_T_	tidal volume
vv-ECMO	venovenous extracorporeal membrane oxygenation
